# Antioxidants in Age-Related Diseases and Anti-Aging Strategies

**DOI:** 10.3390/antiox11101868

**Published:** 2022-09-21

**Authors:** Marius Emil Rusu, Ionel Fizeșan, Laurian Vlase, Daniela-Saveta Popa

**Affiliations:** 1 Department of Pharmaceutical Technology and Biopharmaceutics, Faculty of Pharmacy, Iuliu Hatieganu University of Medicine and Pharmacy, 8 Victor Babes, 400012 Cluj-Napoca, Romania; 2 Department of Toxicology, Faculty of Pharmacy, Iuliu Hatieganu University of Medicine and Pharmacy, 8 Victor Babes, 400012 Cluj-Napoca, Romania

Aging is an intricate process and an important risk factor in the development and advancement of many disorders. As the world’s population ages, chronic diseases associated with age will become increasingly common with a strong impact on quality of life. The pathogenesis of various diseases, including cardiometabolic disorders, neurodegenerative diseases, and cancer, consists of the accumulation of reactive oxygen species that leads to oxidative stress and inflammaging, which are major contributors to cellular senescence.

Different strategies have been suggested for healthy aging and for delaying or slowing aging. Plant matrices rich in antioxidant molecules, such as polyphenols, phytosterols, vitamins, and minerals, have been revealed to diminish the risk of age-associated syndromes in numerous in vitro studies [1,2]. Based on the fact that in vitro beneficial outcomes of any compound or extract should be confirmed through in vivo toxicological studies [3], the antioxidant and anti-inflammatory effects were replicated in animal model interventions revealing lower levels of reactive oxygen species (ROS), advanced glycation end products, or inflammatory biomarkers [4,5]. Moreover, clinical evidence supported the potential of bioactive compounds in the decrease in many risk factors related to aging and the likely prevention of age-related diseases [6].

This Special Issue “Antioxidants in Age-Related Diseases and Anti-Aging Strategies”, which includes five research articles, four review reports, and one systematic review and meta-analysis, adds new contributions that describe the mechanisms by which oxidative stress and inflammatory factors cause the occurrence or progression of age-related chronic diseases, as well as new strategies to treat or prevent these pathological conditions.

The study by Ghzaiel et al. [7] assayed the chemical composition of *Pistacia lentiscus* L. seed oil (PLSO) in terms of polyphenols, flavonoids, phytosterols, α-tocopherol, β-carotene, and fatty acids and its antioxidant activity. This was followed by an in vitro evaluation of the potential of this plant to neutralize the cytotoxic effects induced by 7β-hydroxycholesterol (7β-OHC) in murine C2C12 myoblasts. 7β-OHC is an oxysterol that can activate oxidative stress and inflammation and contribute to age-related diseases, such as sarcopenia. The results showed that PLSO contains a combination of molecules capable of diminishing 7β-OHC-induced cytotoxic effects and activating cytoprotective properties, including the prevention of cell death and organelle dysfunction, attenuation of oxidative stress, and activity normalization of glutathione peroxidase (GPx) and superoxide dismutase (SOD), which are key antioxidant enzymes. Thus, PLSO can prevent age-related diseases, such as sarcopenia, an age-related gradual deterioration in skeletal muscle mass, strength, and function.

The skin is a barrier between the body and the environment, protecting the body from environmental pollutants and pathogens. One study [8] was conducted to discover a natural product that efficiently mitigates the cytotoxicity and oxidative stress induced by airborne particulate matter with a size of 10 μm or less (PM_10_) in the skin. Data showed that Siegesbeckiae Herba extract (SHE), a hot water extract prepared from dried leaves of *Siegesbeckia pubescens* Makino, had the potential to relieve PM_10_-induced cytotoxicity and increase the cellular antioxidant capacity in HaCaT cells, an immortalized human keratinocyte cell line. SHE mitigated PM_10_-induced cell death, lactate dehydrogenase (LDH) release, and lipid peroxidation and lowered ROS production. Additionally, SHE activated the NRF2 system in cells by decreasing the expression of KEAP1, a negative regulator of NRF2, and increasing the expression of HMOX1 and NQO1, two main target genes of NRF2. Chlorogenic acid may be an active phytochemical in activating the NRF2 pathway. Moreover, SHE selectively induced the enzymes involved in the synthesis and regeneration of reduced glutathione (GSH) and increased the cellular content of GSH, preventing the oxidation of GSH to GSSG triggered by PM_10_ exposure. The findings of this experiment should be verified using normal human epidermal keratinocytes and animal models.

Another study [9] analyzed plant compounds that might mitigate skin wrinkle formation, inflammation, pigmentation, and dehydration caused by chronic irradiation with ultraviolet B (UVB) light. UVB can activate signaling pathways, leading to the upregulation of genes, including matrix metalloproteinases (MMPs), especially MMP-1, involved in collagen degradation and wrinkle formation, and cyclooxygenase 2 (COX-2), involved in skin inflammation and photoaging. *Rosa gallica*, one of the most commonly used Rosa species for culinary, medicinal, and cosmetic purposes, which showed in vitro potential to prevent biomarkers of skin aging, was used in this in vivo study. The gavage administration of *Rosa gallica* extract prevented UVB-mediated skin wrinkle development and collagen degradation in the dorsal skin of female mice by down-regulating UVB-induced COX-2 and MMP-1 expression. Gallic acid was the major molecule contributing to the anti-skin-aging effect by selectively inhibiting c-Raf/MEK/ERK/c-Fos signaling axis. Since molecules targeting the c-Raf pathway exert chemopreventive and chemotherapeutic effects against different cancer types, gallic acid could potentially suppress carcinogenesis. The study concluded that *Rosa gallica* and gallic acid could protect skin cells against free radicals and inflammatory reactions, leading to a reduction in skin aging.

The third study [10] exploring skin health examined the beneficial activities of cyanidin- and malvidin-3-*O*-glucosides, molecules belonging to the anthocyanin family, and some of their structurally related pigments. Most of the examined compounds were found to reduce biofilm production by *Staphylococcus aureus* and *Pseudomonas aeruginosa*, displayed UV-filter capacity, and also reduced the production of ROS in human skin epidermal keratinocytes and dermal fibroblast. Furthermore, the molecules revealed inhibitory activity of skin-degrading enzymes, hyaluronidase, collagenase, and elastase, the three main enzymes responsible for the regulation of the structural integrity of skin layers, showing no significant cytotoxicity. The stability issue of anthocyanin family compounds can be overcome by the use of anthocyanin’s structural derivatives, such as carboxypyranocyanidin-3-*O*-glucoside, a molecule with great structural stability that could be included in topical formulations for cosmeceutical purposes.

In their study, Muraleva et al. [11] investigated the effects of mitochondria-targeted antioxidant plastoquinonyl-decyltriphenylphosphonium (SkQ1) in MEK1/2-ERK pathway alterations as a therapeutic target in Alzheimer’s disease (AD). MEK1 and MEK2 are tyrosine/threonine protein kinases found in the Ras/Raf/MEK/ERK mitogen-activated protein kinase (MAPK) signaling pathway. The study included senescence-accelerated OXYS rats that develop neurodegenerative changes almost similar (>90% of cases) to the signs of sporadic AD in humans. The results showed that, compared to untreated control (Wistar) rats, SkQ1 eliminated differences in the expression of eight out of nine genes involved in the hippocampal extracellular regulated kinases’ (ERK1 and -2) signaling pathways. Additionally, SkQ1 reduced the hyperphosphorylation of tau protein that is present in pathological aggregates in AD. Thus, SkQ1 alleviates AD pathology in the OXYS rat hippocampus by reducing MEK1/2-ERK1/2 phosphorylation and may be a promising drug for human AD.

Collins et al. [12] presented a thorough revision of antioxidant therapy in AD. Strong evidence supports the role of oxidative stress in the pathogenesis and progression of AD and aging. Oxidative stress appears when there is an imbalance among antioxidants and the production and buildup of ROS, related to an insufficient or dysfunctional antioxidant defense system with damaging effects to important cellular structures including proteins, lipids, and nucleic acids. Adequate cellular homeostasis, a balance between the production and depletion of ROS, occurs through the protective mechanisms of natural and synthetic antioxidants. Natural antioxidants can be further divided into enzymatic antioxidants, enzymes produced in the body with free-radical-scavenging abilities, such as SOD, catalase (CAT), GPx, glutathione reductase (GR), and glucose-6-phosphate dehydrogenase (G6PDH) and non-enzymatic antioxidants, including polyphenols, carotenoids, vitamins, and minerals.

Although available medications help with AD symptom management, there are no treatments to prevent or cure the disease, and furthermore, none of the currently existing treatments address oxidative stress. Thus, recent studies focused on the use of antioxidants to diminish the oxidative stress effects on the central nervous system. In preclinical in vitro and in vivo experiments, a combination of antioxidant compounds improved the overall antioxidant capacity of drug therapy, enhanced the bioavailability to various cellular locations, and increased the functionality of antioxidant molecules. The therapeutic potential of natural antioxidants in preventing and/or treating neurodegenerative conditions is presently assessed in human clinical trials.

The biological activities and cosmeceutical properties of nicotinamide were discussed in an interesting review [13]. Nicotinamide (niacinamide) is mostly used as a nutritional supplement for vitamin B3 (nicotinic acid, niacin), and vitamin B3 is further used in the synthesis of the NAD^+^ family of coenzymes, related to cellular energy metabolism and defense systems. Nicotinamide supplementation restores mitochondrial energetics and cellular NAD^+^ pool, prevents the skin pigmentation process, and diminishes oxidative stress and inflammatory response. This molecule has the potential to support skin homeostasis by regulating the cellular redox status. In clinical trials, topically applied nicotinamide was well tolerated by the skin and reduced hyperpigmentation and the progression of skin aging. Therefore, it may be useful as a cosmeceutical ingredient to attenuate skin aging, especially in the elderly or in patients with reduced NAD^+^ pool in the skin.

Another review [14] examined vitamin K and its role as a vital cofactor in the activation of several proteins, which act against age-related diseases. It was shown that vitamin K carboxylates osteocalcin, a protein responsible for transporting and fixing calcium in bones, activates matrix Gla protein, an inhibitor of vascular calcification and cardiovascular incidents; carboxylates Gas6 protein, which is involved in the physiology of the brain and may inhibit cognitive decline and neurodegenerative disease; and improves insulin sensitivity, thus decreasing diabetes risk. Additionally, vitamin K presents antiproliferative, proapoptotic, and autophagic effects and is associated with reduced cancer risk. Recent evidence indicates that protein S, another vitamin K-dependent protein, might prevent the cytokine storm noticed in COVID-19 cases. The latest scientific documents emphasize the vitamin K role in preventing age-associated diseases and improving the efficiency of medical treatments in the elderly.

In a narrative review, Barbalho et al. [15] analyzed *Ginkgo biloba* (GB), a medicinal plant in the Ginkgoaceae family considered to be the oldest tree alive in the world. GB extracts account for some health benefits for memory and cognition, AD, Parkinson’s disease (PD), and dementia, which are attributed to its antioxidant, anti-inflammatory, and antiapoptotic activities. In addition, GB can exert benefits in cardiovascular conditions, hypertension, insulin resistance, fasting serum glucose, glycated hemoglobin, and dyslipidemia. Moreover, it can improve cerebral blood flow supply, executive function, attention/concentration, and non-verbal memory and decrease stress. In many European states, GB extract is the only drug therapy for the treatment of mild cognitive impairment. The bioactive compounds, mainly polyphenols, flavonoids, terpenoids, and organic acids, are responsible for the beneficial effects. This review revealed that GB could be considered in the therapeutic and preventative methods for aging-associated conditions and the aging process. However, more studies are required to determine the doses, pharmaceutical form, and treatment time needed in aging conditions.

A systematic review and meta-analysis [16] investigated biomarkers of metabolic syndrome and inflammation as pathophysiological predictors of senescence and age-related diseases. Recent evidence confirmed that particular diets rich in antioxidant bioactive compounds and a balanced lipid profile, such as walnuts, could have beneficial results on human health. A systematic search in several databases was performed to find randomized controlled trials reporting on the outcomes of walnut consumption on metabolic syndrome and inflammatory markers in middle-aged and older adults. The investigation extracted 17 studies, including 11 crossover and 6 parallel trials. The analysis revealed that walnut-enriched diets had significant reducing effects on triglyceride, total cholesterol, LDL cholesterol levels, and some inflammatory markers, with no adverse effects on anthropometric and glycemic parameters. While further and better-designed reports are required, the outcomes stress the benefits of including walnuts in the dietary plans of middle-aged and older adults.

We would like to acknowledge all the authors who have contributed to this Special Issue and have provided a better understanding of the importance of antioxidants in the delaying of aging and prevention of diseases associated with aging, and the need to develop future therapeutic strategies targeting these diseases.
